# Comparative study of Co_3_O_4_(111), CoFe_2_O_4_(111), and Fe_3_O_4_(111) thin film electrocatalysts for the oxygen evolution reaction

**DOI:** 10.1038/s41467-023-40461-0

**Published:** 2023-08-08

**Authors:** Earl Matthew Davis, Arno Bergmann, Chao Zhan, Helmut Kuhlenbeck, Beatriz Roldan Cuenya

**Affiliations:** Department of Interface Science, Fritz-Haber Institute of the Max Planck Society, 14195 Berlin, Germany

**Keywords:** Electrocatalysis, Surface spectroscopy, Scanning probe microscopy

## Abstract

Water electrolysis to produce ‘green H_2_’ with renewable energy is a promising option for the upcoming green economy. However, the slow and complex oxygen evolution reaction at the anode limits the efficiency. Co_3_O_4_ with added iron is a capable catalyst for this reaction, but the role of iron is presently unclear. To investigate this topic, we compare epitaxial Co_3_O_4_(111), CoFe_2_O_4_(111), and Fe_3_O_4_(111) thin film model electrocatalysts, combining quasi in-situ preparation and characterization in ultra-high vacuum with electrochemistry experiments. The well-defined composition and structure of the thin epitaxial films permits the obtention of quantitatively comparable results. CoFe_2_O_4_(111) is found to be up to about four times more active than Co_3_O_4_(111) and about nine times more than Fe_3_O_4_(111), with the activity depending acutely on the Co/Fe concentration ratio. Under reaction conditions, all three oxides are covered by oxyhydroxide. For CoFe_2_O_4_(111), the oxyhydroxide’s Fe/Co concentration ratio is stabilized by partial iron dissolution.

## Abstract (web summary)

Cobalt-based oxidic anodes with added iron are good electrocatalysts for alkaline oxygen evolution reaction, but the role of iron is still unclear. Here the authors investigate oxygen evolution reaction activity of three well-defined epitaxial thin-film electrodes to address this issue.

## Introduction

Recent years have seen a push towards renewable energy sources in the transition away from our dependence on fossil fuels. However, due to natural fluctuations of sources such as solar, wind, and tidal energy, an efficient energy storage path is needed for situations where the production level does not match the consumption. One potential route for energy storage is the electrocatalytic production of H_2_ via water splitting. H_2_ produced this way is called green hydrogen when the energy stems from renewable sources^1,2^. The limiting factor in this process is the sluggish kinetics of the oxygen evolution reaction (OER) and its high thermodynamic potential.

Catalysts based on noble metals such as Ru and Ir exhibit excellent OER performance in acidic media, but their high cost conflicts with a large-scale use^3^. On the other hand, a number of oxides of less expensive, abundant metals have shown comparable reactivity in alkaline media, particularly oxide spinels containing Co, Ni, and Fe^2,4^. According to current knowledge, the real catalysts are not the oxides themselves, but surface oxyhydroxide layers which form under OER conditions^5–8^. Thus, the oxides are just pre-catalysts. Pure iron oxide was not predicted to yield a good catalyst^9^, but it was discovered that Fe-impurities in the electrolyte could enhance the activity of other oxides and oxyhydroxides^10,11^. This also holds for Co and Ni-based catalysts^12,13^, and it has been proposed that the active sites in iron-containing mixed oxyhydroxide catalysts are iron-based^14–16^. A recent study by Haase et al. reveals the presence of oxyl species on cobalt oxyhydroxide during OER, which may also play a relevant role for the reaction^17^.

Understanding catalytic processes on nanoparticulate ‘real’ catalysts is demanding. For catalysis, the surface structure is relevant, but this can be very complex in such systems, and usually only incomplete knowledge is available. Moreover, the catalyst’s composition and structure are often inhomogeneous, which additionally enhances the complexity. Epitaxial thin film model catalysts are not afflicted by these issues: they have a well-defined surface structure and composition, and all parts of the surface can be equally well reached by the electrolyte. Furthermore, electrical conductivity, and its variation over the surface area can be a problem for the study of nanoparticulate ‘real’ catalysts, whereas homogeneous thin films with a thickness of just a few nanometres suffer much less from this issue even if the film material is nominally insulating. Consequently, results for epitaxial films can be more easily interpreted, and data for different films can be compared quantitatively if the experimental conditions are identical.

Only very few experimental OER studies have been performed on well-ordered surfaces within the Co-Fe-O system. These include Co_3_O_4_(111) and CoOOH(001)^18–20^, CoO_*x*_ nano-islands on Au(111)^21,22^, and Fe_3_O_4_(001) and (110) single crystal surfaces^23,24^. Building on the work of Bergmann et al. on polycrystalline Co_3_O_4_ thin film catalysts^25^, Reikowski et al. elegantly showed that a thin layer of CoOOH is formed on the surface of Co_3_O_4_(111) in a rapid and reversible process that begins before the onset of OER^20^. Chung et al. recently reported that Fe ions in iron oxyhydroxides are “dynamically stable”, i.e., the ions are continuously dissolved from and redeposited onto the surface^26^. Müllner et al. used single crystal Fe_3_O_4_ samples to show that the (001) and (110) surfaces are stable during OER, and that the (110) surface is more reactive^23^. Further, an *operando* study by Grumelli et al. demonstrated that the (√2×√2)R45° surface reconstruction of Fe_3_O_4_(001) is maintained deep into the OER regime and has a large effect on the OER kinetics^24^. Han et al. prepared Co_x_Fe_3-x_O_4_ films with different preferential orientations via electrodeposition on Cu single crystal substrates^27^ and found that the OER activity depends on the surface orientation, with (110) > (111) > (001). The same orientation-reactivity trend was found by Poulain et al. for NiO thin films^6^.

The central topic of the present study is the investigation of the effect of the Co/Fe concentration ratio on the OER activity for films of the same surface orientation^8,13,27,28^. We found the best OER reactivity and stability for mixed Co_1+δ_Fe_2−δ_O_4_(111) layers (|δ| <0.2) with a low Fe concentration, while pure cobalt oxide and iron oxide layers lag behind. However, Co_3_O_4_(111) freshly introduced into the electrolyte had an initial similar activity as the best Co_1+δ_Fe_2__−__δ_O_4_(111) film, pointing towards a high activity of cobalt hydroxide which is unstable under OER conditions. Furthermore, for Co_1+δ_Fe_2−δ_O_4_(111), we observed a reduction of the Fe concentration by Fe dissolution into the electrolyte during OER, accompanied by an increase of the OER activity. Our results reveal that the cobalt/iron ratio is an important parameter for the OER reactivity of the films, while the surface morphology plays only a minor role.

## Results

### Pre- and post-electrochemistry characterization in ultra-high vacuum

**Fig. 1:**
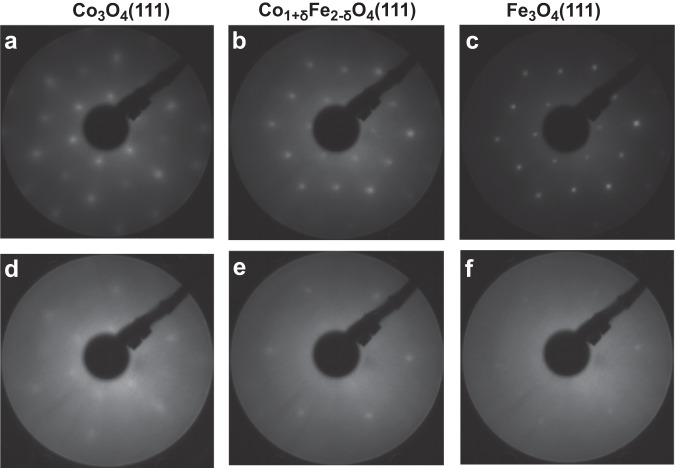
LEED patterns of the thin films. Panels (**a**–**c**) before electrochemistry, (**d**–**f**) after electrochemistry. The electron energy was 160 eV. The  contrast of the images has been digitally enhanced to improve the visibility of the structures.

Well-defined epitaxial thin films were prepared via oxidative physical vapour deposition as discussed in the experimental section. The samples were characterized in situ with X-ray photoelectron spectroscopy (XPS), low-energy electron diffraction (LEED), and scanning tunnelling microscopy (STM) before and after the electrochemistry experiments. After more than 2 h under OER conditions, the oxide films still exhibited a visible LEED pattern, see Fig. 1. The high background intensity after OER indicates a high level of surface disorder, most pronounced for Fe_3_O_4_(111). We assign this to remnants of the (oxy)hydroxide layers formed during OER^5,7,20,25^. They might be disordered and thus be partially responsible for the diffuse LEED background. The XPS data discussed later clearly reveal the presence of such layers.

**Fig. 2:**
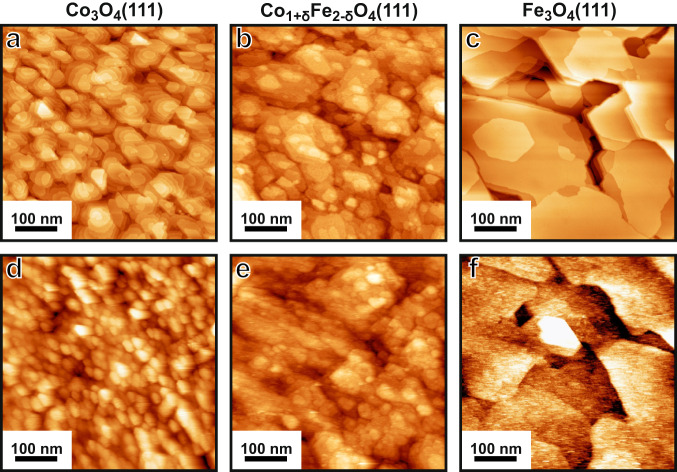
STM images of the thin films. Panels (**a**–**c**) before, and (**d**–**f**) after more than 2 h of electrochemistry. Tunnelling conditions: (**a**–**c**, **e**, **f**) sample bias 2.0 V, current 0.1 nA, (**d**) 3.0 V, 0.2 nA.

The STM images in Fig. 2a–c show the general surface morphology of the as-prepared Co_3_O_4_(111), Fe_3_O_4_(111) and Co_1+δ_Fe_2−δ_O_4_(111) films. The step height of the terraces on all three films is ~4.8 Å, which is the distance between equivalent layers along [111]. The Fe_3_O_4_(111) film has terraces larger than 100 nm across, while the Co_1+δ_Fe_2−δ_O_4_(111) terraces are smaller, approximately 20–60 nm wide. Co_3_O_4_(111) exhibits an island-like structure, with terrace widths in the range of 10–30 nm. RMS roughness values are listed in Table 1. The presence of steps and edges gives rise to an increased surface area, and therefore, the areas of the surface profiles are larger than the STM scan areas. The ratio of both (determined using the WSXM software^29^ from several 500 × 500 nm STM images) is the scaling or roughness factor listed in Table 1. By multiplying the area enclosed by the electrochemical cell (0.283 cm^2^) by the scaling factor we can get an estimate of the electrochemically active surface area (ECSA) and find that the ECSA is not much different between the different as-prepared films.

**Table 1:** Root mean square (RMS) roughness, surface area scaling factor, electrochemical surface area (ECSA), and specific capacitance, C_s_, for Co_3_O_4_(111), Co_1+δ_Fe_2−δ_O_4_(111), and Fe_3_O_4_(111) before and after OER (oxygen evolution reaction)

	Before OER				After OER			
Film	RMS (nm)	Surface area scaling factor	ECSA (cm^2^)	*C*_s_ (mF/cm^2^)	RMS (nm)	Surface area scaling factor	ECSA (cm^2^)	*C*_s_ (mF/cm^2^)
Co_3_O_4_(111)	1.56	1.04	0.294	0.082 ± 0.01	2.21	1.07	0.302	0.079 ± 0.01
Co_1+δ_Fe_2−δ_O_4_(111)	1.09	1.03	0.291	0.069 ± 0.01	0.99	1.07	0.302	0.073 ± 0.01
Fe_3_O_4_(111)	0.75	1.01	0.286	0.087 ± 0.02	0.70	1.16	0.330	0.061 ± 0.01

The C_s_ capacitances reported before and after OER are computed from double-layer charges determined with potentiostatic electrochemical impedance spectroscopy (PEIS) at the beginning and the end of a 2-h chronoamperometry (CA) run at OER conditions.

In order to determine the specific capacitance, C_s_^30^, we divided the double-layer capacitance measured with potentiostatic electrochemical impedance spectroscopy (PEIS) (Supplementary Fig. S1 and Supplementary Table S1) by the ECSA listed in Table 1. Interestingly, the values of C_s_ for the Co_3_O_4_(111) surface are ~3 times larger than those determined for metallic Co^30^, which may be due to different surface structures and oxidation states.

An investigation of the Co_1+δ_Fe_2−δ_O_4_(111) surface structure has not yet been reported. Quantitative XPS analysis finds an enhanced iron concentration in the surface-sensitive spectra for as-prepared Co_1+δ_Fe_2−δ_O_4_(111) (see Supplementary Fig. S2), indicating that the terminating layer consists mostly of iron. In agreement with that, atomic resolution STM images (panels d,e of Supplementary Fig. S3) exhibit similar arrangements of protrusions for Fe_3_O_4_(111) and Co_1+δ_Fe_2−δ_O_4_(111). Thus, the combined information from STM, LEED, and XPS infers that tetrahedrally terminated Fe_3_O_4_(111) terminates most of the Co_1+δ_Fe_2−δ_O_4_(111) surface (Supplementary Fig. S4). This is plausible, since Co_3_O_4_(111) and Fe_3_O_4_(111) are both terminated by tetrahedral layers^31–33^, and Co_1+δ_Fe_2−δ_O_4_(111) might share this termination, being a mixture of both.

Comparison of the Fe_3_O_4_(111) and Co_1+δ_Fe_2−δ_O_4_(111) STM images recorded before and after electrochemistry (Fig. 2) reveals that the terrace structures are not significantly modified, as also reflected in the small changes in the RMS roughness (see Table 1). The latter gets slightly smaller, indicating that the OER remnants tend to smoothen the surface by filling the valleys. On the other hand, the Co_3_O_4_(111) surface is more granular after electrochemistry: the density of islands has approximately doubled, and the RMS roughness is ~40% larger (Table 1). After electrochemistry, there is clearly an enhanced short-scale roughness for Fe_3_O_4_(111) on the terraces, accompanied by a notable surface area increase and a C_s_ decrease. This may be related to the oxidation of the film (see discussion below) or structural rearrangements during the formation of the oxyhydroxide layer.

**Fig. 3:**
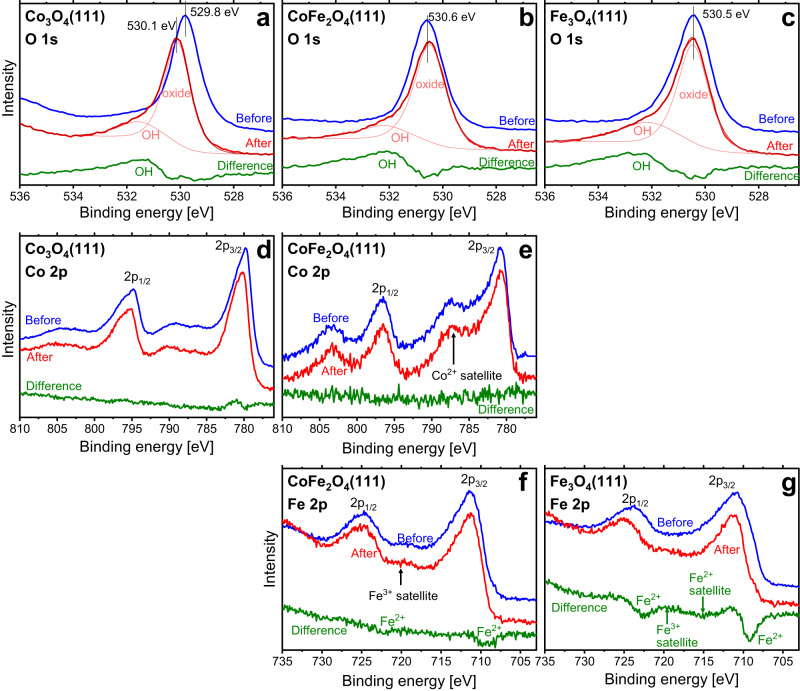
X-ray photoelectron spectroscopy (XPS) spectra from the O 1*s* and metal 2p regions recorded before (blue lines) and after (red lines) electrochemistry (offset for clarity). Panels (**a**, **d**): Co_3_O_4_(111), (**b**, **e**, **f**): Co_1+δ_Fe_2−δ_O_4_(111), (**c**, **g**): Fe_3_O_4_(111). Difference spectra (‘After’–‘Before’) are plotted using olive lines. Peak fits for the O 1s spectra after electrochemistry are shown as thin red and brown lines. Prior to the calculation of the 2*p* difference spectra, the 2*p* spectra recorded before electrochemistry were shifted on the energy axis (to compensate for band bending effects) to align them with the spectra recorded after electrochemistry and they were normalized to identical peak areas to reveal changes of the electronic structure. Therefore, concentration changes are masked in the different data.

O 1*s* and metal 2*p* XPS spectra are compiled in Fig. 3. The O 1*s* spectra of all three films (panels a-c) show the main oxide peak at ~530 eV and after electrochemistry an additional small peak at about 1.5 eV higher binding energy, which we assign to surface hydroxide. Oxyhydroxide O1*s* peaks overlap with the oxide bulk peak and are therefore not clearly detectable^34^. Yang et al. identified the cobalt hydroxide O 1*s* bulk peak at 531.2 eV, somewhere between the surface hydroxide and the bulk oxide peak^35^, which makes it hard to clearly identify it. Since the layer may also contain oxyhydroxide, we use the notation ‘(oxy)hydroxide’ when we refer to the full layer. Assuming a flat and homogeneous layer, we find a surface (oxy)hydroxide layer thickness in the range of 3 Å for all three oxides. The whole surface layer may not be much thicker, as concluded from the intensity of the LEED spots after OER (see Fig. 1), which would be compatible with a flat layer less than ~1 nm thick (for details see SI). For Fe_3_O_4_(111) it was checked whether the hydroxide stems from the electrochemical experiments by recording an O 1*s* spectrum from a sample area not exposed to the electrolyte (Supplementary Fig. S5). The much weaker hydroxide-related O 1*s* intensity shows that the hydroxide layer indeed results mostly from the OER electrochemistry. It is expected that the same holds for the Co_3_O_4_(111) and Co_1+δ_Fe_2−δ_O_4_(111) samples.

Contaminants were absent according to the XPS spectra, except for a minute amount of adventitious carbon (<1 monolayer). Substrate material (Pt or Au) could not be detected (Supplementary Fig. S6), indicating that all films are closed.

The Fe 2*p* spectra of the pure Fe_3_O_4_(111) film, Fig. 3g, reveal a strongly reduced Fe^2+^ intensity after OER. This will (in part) be due to oxyhydroxide formed during OER, but in view of the limited thickness of the layer, one may expect that also part of the oxide becomes oxidized to Fe_2_O_3_. Such an oxidation process has also been observed for Fe_3_O_4_(100) by Müllner et al.^23^. and for Fe_3_O_4_ nanocubes by Hsu et al.^36^. The Fe_3_O_4_(111) LEED pattern of the oxide after OER shows spots at similar positions as before OER, see Fig. 1c, f, which would be compatible with the formation of maghemite (γ-Fe_2_O_3_), which is cubic and can be formed by dissolution of tetrahedrally-coordinated iron out of the Fe_3_O_4_ lattice leading to a Fe oxidation state of 3+ without changing the lattice structure. As shown in Table 1, the specific capacitance of the Fe_3_O_4_(111) film has decreased by ~25 %, which we attribute to the oxidation process.

For Co_3_O_4_(111), the Co 2*p* spectrum (panel d) is very similar to spectra reported by others^37^ and is not affected very much by the OER process, which means that the oxide is mostly stable under the chosen OER conditions. The features in the difference spectrum at around 780 eV are consistent with the formation of CoOOH at the surface^37,38^. Likewise, the small shift of the main lines to slightly higher binding energy would be compatible with the formation of CoOOH and Co(OH)_2_^35^.

For the Co_1+δ_Fe_2−δ_O_4_(111) film, the Co 2*p* spectra recorded before and after electrochemistry (panel e) are also very similar, with very small CoOOH-related features in the region around 780 eV. Within the composition range |δ| <=0.2, the spectra do not vary much and therefore only data for a selected film (Film 1, Table 2) are shown. Clearly, the Co 2*p* spectrum of Co_1+δ_Fe_2−δ_O_4_(111) is different from the corresponding Co_3_O_4_(111) spectrum in panel d, which results from the fact that the cobalt ions in Co_1+δ_Fe_2−δ_O_4_(111) are in a 2+ oxidation state, and therefore, the Co 2*p* spectrum is similar to that of CoO^37^.

**Table 2:** Fe concentrations (the cobalt concentration is C_Co_ = 100-C_Fe_) as a percentage of the total metal content for the prepared Co_1+δ_Fe_2−δ_O_4_(111) films before and after oxygen evolution reaction (OER) experiments

		*θ* = 0°	*θ* = 70°	*θ* = 0°	*θ* = 70°	*θ* = 0°	*θ* = 70°
No	δ before OER	Fe concentration before OER		Fe concentration after OER		Difference (‘after’-‘before’)	
1	0.20	60%	64%	52%	49%	8%	15%
2	0.08	64%	70%	55%	48%	9%	22%
3	0.05	65%	70%	not measured	not measured		
4	0.02	66%	70%	58%	53%	8%	17%
5	−0.04	68%	76%	62%	62%	6%	14%

Concentrations are calculated from the areas of the Fe 2*p* and Co 2*p* peaks in the XPS spectra (see Fig. 3). The electron detection angles θ are given relative to the surface normal. The XPS sampling depths are: λ = 12.0 Å and 4.1 Å for Fe and 10.9 Å and 3.7 Å for Co, considering 0° and 70° as respective detection angles. For more details, see the SI.

A quantitative evaluation of the Co 2*p* and Fe 2*p* spectra of the Co_1+δ_Fe_2−δ_O_4_(111) films reveals a decrease of the Fe concentration during electrochemistry in all experiments. Table 2 shows that the decrease is more pronounced in the surface-sensitive spectra, which demonstrates that the iron loss occurs at the surface.

No significant changes of the Co_1+δ_Fe_2−δ_O_4_(111) surface compositions were observed when the samples were placed in the electrolyte without applied potential, which shows that the iron loss only occurs under OER conditions (see Supplementary Fig. S7). An OER run performed for 10 min instead of 2 h showed a similar loss, indicating that it occurred mostly within the first 10 min. However, there are probably also some long-term losses as indicated by Supplementary Fig. S8, which reveals an activity improvement for more than 20 h.

The Fe^2+^ intensity of Co_1+δ_Fe_2−δ_O_4_(111) is reduced after OER, revealing that part of the surface iron is oxidized. Oxyhydroxide formation and oxidation of the iron oxide at the surface may be responsible for this. The Co^2+^ ions are apparently not further oxidized under the given OER conditions, similar to the observation for Co_3_O_4_(111).

### Electrochemical measurements

**Fig. 4:**
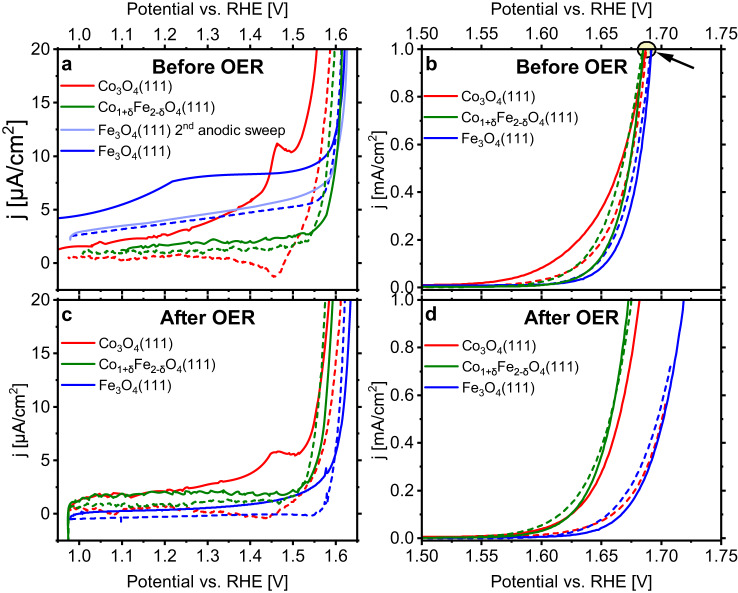
Cyclic voltammograms of Co_3_O_4_(111), Fe_3_O_4_(111), and Co_1+δ_Fe_2−δ_O_4_(111) thin films. The data in (**a**, **b**) were recorded at the beginning of the experiment from the open-circuit voltage (OCV) up to 1 mA/cm^2^, and the data in (**c**, **d**) after 2 h at OER (oxygen evolution reaction) conditions starting with a cathodic sweep. Panels (**a**) and (**c**) show the redox transition region. In (**a**) the second anodic sweep of the Fe_3_O_4_(111) sample is displayed to show the disappearance of the redox transition at ~1.26 V. Scan rate: 5 mV/s. Electrolyte: 0.1 M KOH. Cathodic scans are shown as dashed lines. The arrow in panel (**b**) marks the potentials where the current densities reach 1 mA/cm^2^. These potentials were employed for the subsequent CA measurements. For Co_1+δ_Fe_2−δ_O_4_(111), data from the film with the lowest Fe concentration are shown [Film 1, Table 2]. The resistances for the IR correction and the high-frequency cell resistances are listed in Supplementary Tables S2 and S3, respectively.

Cyclic voltammetry (CV) was applied to identify the influence of Fe on the surface redox electrochemistry. Before a 2-h OER run, linear sweep voltammograms (LSVs) were recorded from the open-circuit voltage (OCV) up to OER conditions (defined here as the potential where the current density reaches 1 mA/cm^2^), see Fig. 4a, b.

For the Co_3_O_4_(111) film, a redox feature is visible at ~1.46 V. This feature has typically been assigned to a Co^3+^/Co^4+^ redox transition^39,40^. The assignment is likely a strong simplification as it neglects metal-ligand charge reorganization leading to electron–hole formation at surface oxygen ions, similar to IrO_x_^17,41^. There is no indication of a peak previously reported between 1.0 and 1.3 V for other Co-based electrocatalysts^18,25,42^. We note that a peak at this potential has been observed more frequently for layered CoOOH-like structures than for Co_3_O_4_-like oxides^25^.

The first anodic linear sweep of the Fe_3_O_4_(111) film exhibits broad oxidation features centred at approximately 0.73 V (Supplementary Fig. S9) and 1.26 V vs RHE. These features only appear in the first sweep, and no reduction is observed during the following cathodic scan, nor further oxidation on later anodic sweeps up to OER conditions. We tentatively assign these transitions to the oxidation of near-surface iron atoms in the context of the Fe_3_O_4_ → Fe_2_O_3_ transformation. These redox features were completely missing in the Fe_3_O_4_(001) CV data published by Grumelli et al.^24^. However, Calvillo et al. observed peaks at ~1.25 V during the first two CVs for Fe_3_O_4_/Pd(001) films^43^.

An approximately linear offset is seen in the initial Fe_3_O_4_(111) CVs (see Supplementary Fig. S9). The background current observed may be due to a slow oxidation process in the bulk of the film. In the CVs obtained after OER this background is not present anymore, which is another indication that it is related to the Fe_3_O_4_ → Fe_2_O_3_ transformation, since the oxidation process may be largely complete or have slowed down after OER.

The initial anodic scan of the Co_1+δ_Fe_2−δ_O_4_(111) films had no distinguishable redox features. This may be because the Fe in Co_1+δ_Fe_2−δ_O_4_ is already in a 3+ oxidation state^44^, which is probably also the case for most of the surface iron atoms (there is only a small Fe^2+^ contribution, see Fig. 3f). As discussed, the cobalt concentration at the surface of the pristine sample is probably small, which could explain why Co redox transitions were not detected. The peak expected between 1.0 and 1.3 V_RHE_ may be absent for a similar reason as discussed for Co_3_O_4_(111). Additionally, the presence of Fe has been shown to shift the Co oxidation peaks to higher potentials^28^. Therefore, the peak that we observe at 1.46 V for Co_3_O_4_(111) may be obscured by the onset of OER if it does exist for Co_1+δ_Fe_2−δ_O_4_(111).

After the initial CVs, the samples were exposed to OER conditions for 2 h using the potential where the current density reached 1 mA/cm^2^ in the preceding CV scan (see arrow mark in Fig. 4b). During the 2-h period at OER conditions, chronoamperometry (CA) data were recorded to reveal the time dependence of the current, see Supplementary Fig. S10. Following OER, further CVs were measured, which are shown in Fig. 4c, d. These data reveal that the onset of the Fe_3_O_4_(111) activity after OER is at a significantly higher potential than before OER. Co_3_O_4_(111) has a similar onset potential to Fe_3_O_4_(111) in the cathodic scan after OER, but is much better in the subsequent anodic scan, while the Co_1+δ_Fe_2−δ_O_4_(111) film has a lower onset potential after OER.

**Fig. 5:**
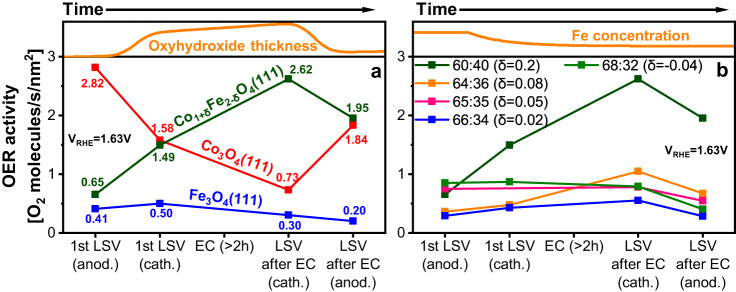
Oxygen evolution activies of the thin films at different stages of the experiment. Panels (**a**) Co_3_O_4_(111), Co_1+δ_Fe_2−δ_O_4_(111), and Fe_3_O_4_(111) thin films,  (**b**) Co_1+δ_Fe_2−δ_O_4_(111) thin films with differing Fe:Co ratios. Data for the Co_1+δ_Fe_2−δ_O_4_(111) film with the lowest Fe concentration (δ = 0.2) are shown in (**a**). The oxyhydroxide layer thickness plot in the top panel in (**a**) and the Fe concentration plot in the top panel in (**b**) are qualitative guesses based on a report that the oxyhydroxide layer thickness increases with increasing potential^20^ and the observation of a decreasing Fe concentration during OER in this study, respectively. ‘anod.’ means ‘anodic’ and ‘cath.’ means ‘cathodic’, both describing the scan direction. The activities are given as the number of O_2_ molecules per second and square nanometre for V_RHE_ = 1.63 V.

OER activity data for a potential of 1.63 V_RHE_ are compiled in Fig. 5 for the thin film oxides using data from linear sweep voltammograms measured at various stages of the experiment. We note that the OER activity was calculated taking into account the surface roughness and thus, correspond to the intrinsic activity of the exposed surfaces. The experimental first step was a CV cycle (an anodic LSV from open circuit potential to OER potential, followed by a cathodic LSV to 1 V_RHE_). Following this, the potential was brought back to that for *j* = 1 mA/cm^2^ and held for at least 2 h. Then, a further CV scan was performed (a cathodic LSV from the potential applied during OER down to 1 V_RHE_, followed by an anodic sweep back to OER conditions). Figure 5 displays the activities derived from the current densities at *V*_RHE_ = 1.63 V during the first CV and the CV after 2 h at OER conditions. Thus, Fig. 5 illustrates condition- and time-dependent trends in the activity of the catalysts. The activity data can be quantitatively compared among the thin films, since the electrochemistry experiments were performed under identical conditions and the ECSAs are well known, see Table 1. Since the data shown at the third abscissa point in Fig. 5, ‘LSV after EC (cathodic)’ were obtained directly after the extended OER scan, they are likely the best representation of the activities of the catalysts under reaction conditions. In panel a, Co_1+δ_Fe_2−δ_O_4_(111) (δ = 0.2) was the most active catalyst at this point. We also found a clear trend in the dependence of the activity on the Fe concentration in Co_1+δ_Fe_2−δ_O_4_(111) in the given concentration range: films with a lower Fe concentration have the higher activity, see panel b. The dependence is significant—the activity varies by several times with a change in Fe concentration of only a few percent.

Under OER conditions, the iron oxide film is covered by an FeOOH layer^7^, which is soluble in basic media, and dissolves slowly during OER, with the rate of dissolution depending on the potential applied^45^. At high anodic potentials it is rapidly oxidized to FeO_4_^2−^, which is highly reactive but also highly soluble^45^. An ICP-MS (inductively coupled plasma mass-spectrometry) characterization of the iron content in the electrolyte revealed a slight increase after electrochemistry, which corresponds to the dissolution of just 0.4 nm of Fe_3_O_4_. This is a negligible part of the Fe_3_O_4_(111) film, which had a thickness between 10 and 15 nm. The data in Fig. 5 also revealed that the activity of Fe_3_O_4_(111) is the lowest of the three catalysts. After a brief improvement at the beginning, it degrades until it finally reaches about 50% of the starting activity. This degradation may in part be due to the oxidation towards Fe_2_O_3_, which is an electrical insulator, but the fact that the activity is the smallest at all abscissa points in Fig. 5 suggests that the oxyhydroxide layer on Fe_3_O_4_(111) simply has a low OER activity, as also proposed by other authors^9^.

To obtain insights into the state of the thin films during OER, *operando* Raman spectra were recorded. The spectra support the XPS result that part of the film transforms to γ-Fe_2_O_3_ during OER (Supplementary Fig. S11 and accompanying discussion). No (oxy)hydroxide-related peaks could be found in any of the Raman spectra even after extended times (>1 h) under OER conditions. This indicates that the oxyhydroxide film was too thin for a detectable signal. *Operando* X-ray scattering was also applied, but also here, the oxyhydroxide could not be clearly detected. This is in line with the results of Wiegmann et al.^46^. who found that the oxyhydroxide layer formed on an Co_3_O_4_(111) layer grown in ultra-high vacuum (UHV) is very thin, thinner than on electrochemically grown Co_3_O_4_(111) layers. It may be assumed that structural imperfections in the latter give rise to this difference.

Figure 5a suggests that the formation and conditioning of the oxyhydroxide layer on Co_3_O_4_(111) has a detrimental effect on the activity: before electrochemistry, the Co_3_O_4_(111) activity is higher than that of the other oxides, but the activity decreases once OER conditions have been reached and it keeps decreasing with increasing OER time. This is also visible in Supplementary Fig. S10, which shows that the Co_3_O_4_(111) OER current density decreases continuously after the start of the experiment. During the cathodic scan after the EC experiments, the activity is as small as that of Fe_3_O_4_(111) while the Co_3_O_4_(111) OER activity improves again when the potential is decreased and the film exposed to reducing conditions, see the last data point in Fig. 5a. One possible conclusion from this observation is that the activity of the surface layer, probably mostly hydroxide, which forms after exposure of the sample to the electrolyte^47^ is higher than that of the thermodynamically more stable oxyhydroxide layer which forms under OER reaction conditions.

An oxyhydroxide layer with an initially high iron concentration may form on Co_1+δ_Fe_2−δ_O_4_(111) due to oxide’s high surface iron concentration, which may be the reason for the similar activities of Fe_3_O_4_(111) and Co_1+δ_Fe_2−δ_O_4_(111) in the initial phase of the experiment, Fig. 5, and also for the similar slopes of the Fe_3_O_4_(111) and Co_1+δ_Fe_2−δ_O_4_(111) CA curves in the first ~50 s, see Supplementary Fig. S10. Beyond this time, the Fe_3_O_4_(111) and Co_1+δ_Fe_2−δ_O_4_(111) CA curves deviate more and more with increasing time. Burke et al. found that (Co,Fe)OOH mixed oxyhydroxide electrodes dissolve in a KOH electrolyte during OER when the Fe concentration is larger than 54%, but they are stable at lower Fe concentrations^28^. We find a reduction of the surface Fe concentration to similar values, see Table 2 for Co_1+δ_Fe_2−δ_O_4_(111) after OER. An ICP-MS characterization of the electrolyte after OER showed the same Fe loss as for the Fe_3_O_4_(111) film (i.e., ~0.4 nm), but no increase of the Co concentration in the electrolyte, indicating that Co is not lost from the film. We hypothesize that iron dissolves into the electrolyte from the oxyhydroxide layer without re-deposition. To test this hypothesis, a Co_1+δ_Fe_2−δ_O_4_(111) film was prepared, but in the final preparation step, pure Fe was deposited instead of a Fe/Co mixture. After oxidizing the film, the excess Fe was still not well-mixed within the film, and it remained as an Fe_3_O_4_(111)-like layer on the surface. A long-term OER CA scan (~21 h, see Supplementary Fig. S8) revealed that for about 1 h the film behaved similar to Fe_3_O_4_(111), where the current mostly decreases as a function of time, see Supplementary Fig. S10. However, after this the current increased, which we attribute to iron dissolution and the concomitant enrichment of cobalt in the oxyhydroxide layer. The Fe concentration following the CA scan was ~50%. Details are discussed in the SI (Supplementary Fig. S8).

Summing up, we have performed a comparative study of the OER activity of epitaxial crystalline thin oxide film model catalysts: Co_3_O_4_(111), Co_1+δ_Fe_2−δ_O_4_(111) (|δ| <=0.2), and Fe_3_O_4_(111). Epitaxial, well-characterized and defect-poor UHV-grown cobalt ferrite layers are used here for the first time for OER studies and permit to quantitatively assess the effect of the iron concentration on the reactivity, which may be a topic for future studies.

Co_3_O_4_(111) and Co_1+δ_Fe_2−δ_O_4_(111) were largely stable under the chosen OER conditions, but Fe_3_O_4_(111) was found to partially transform to the cubic γ-Fe_2_O_3_ phase. Co_1+δ_Fe_2−δ_O_4_(111), the most active catalyst, was found to lose iron during OER via dissolution, eventually converging towards a stable low Fe concentration, while cobalt dissolution was not detected. The Co_3_O_4_(111) surface appears to be somewhat granular after 2 h of OER, while the surface island structures of Co_1+δ_Fe_2−δ_O_4_(111) and Fe_3_O_4_(111) remained nearly unchanged.

Co_3_O_4_(111) has an initial high OER activity which decreases when the OER reaction is running, which we attribute to a higher activity of the surface layer formed after introduction into the electrolyte comprising a higher density of reduced Co^2+^ sites and hydroxyls. In contrast, the Co_1+δ_Fe_2−δ_O_4_(111) activity increases during exposure to OER conditions and decreases upon exposure to reducing conditions. We attribute this finding to the compositional adaptations leading to elevated Fe surface concentration passivating the surface.

We feel that the most important aspect of the thin-epitaxial-film approach is a significant reduction of uncertainties inherent to less well-defined pre-catalyst systems, which permits quantitative comparison of the specific activity for different oxides. Given the homogeneity of the surfaces, we can use laterally averaging spectroscopy methods without averaging over differing areas. These data will constitute a feedstock for theoretical modelling which is likely needed to further unravel mechanistic details of the reaction process but also require more advanced surface sensitive chemical spectroscopy. Upcoming studies will also involve the effect of the facet terminating the surface and thereby, provide further needed insights to yield in a comprehensive picture of the oxygen evolution surface of Co-based electrocatalysts for alkaline water splitting.

## Methods

### Experimental chamber

An UHV chamber with a base pressure of 4 × 10^−11^ mbar was used for sample preparation and surface characterization with XPS, LEED, and STM at room temperature. For some measurements the surface sensitivity of XPS was enhanced by measuring at non-normal detection angles. Unless stated otherwise, measurements were made at normal emission geometry (0°) using Mg Kα radiation (1253.6 eV). Further details of the UHV chamber may be found in the supporting information.

### Thin film preparation

We have developed recipes for the production of well-ordered epitaxial thin films of Co_1+δ_Fe_2−δ_O_4_(111) on Pt(111) and Co_3_O_4_(111) on Au(111). A well-established preparation procedure was employed for Fe_3_O_4_(111) films on Pt(111)^48^. All samples were prepared and characterized quasi in situ in UHV before and after OER without exposure to air at any point to avoid surface contaminations. ‘Quasi in-situ’ means here that all experimental steps were done in the same system, but in different interconnected chambers and environments (UHV, liquid, 1 bar Ar), without intermediate exposure to air during the sample transfer.

The Pt(111) and Au(111) crystal substrates (MaTeck GmbH, Germany) were prepared through cycles of Ar^+^-sputtering and annealing in UHV until a sharp LEED pattern with a low background intensity was observed and no traces of carbon were detected with XPS. Fe and Co were deposited using e-beam assisted evaporators (EFM 4, Omicron) with the deposition rates being calibrated with a quartz microbalance. Deposition rates of 1.2 Å/min for Fe and 0.6 Å/min for Co were employed, except for the growth of Co_3_O_4_(111), where a Co deposition rate of 1.3 Å/min was used.

The Fe_3_O_4_(111) film was grown on a Pt(111) substrate by molecular beam epitaxy (MBE) using a slightly modified version of the procedure described by Sala et al.^48^. First, 4.1 Å of Fe was deposited at room temperature. The sample was then heated in 1 × 10^−6^ mbar O_2_ from room temperature up to 1000 K, where it was held for 2 min. The O_2_ pressure was not reduced until the sample had cooled down to 500 K after annealing. This led to the formation of an FeO(111) layer covering the whole substrate. Then, in at least 3 cycles, ~20 Å of Fe were deposited at room temperature followed by annealing in 5 × 10^−6^ mbar of O_2_ for 20 min. This led to films approximately 10–15 nm thick. The oxidation temperature was limited to 850 K to avoid de-wetting of the film. This limited temperature resulted in broader LEED spots and smaller terraces, but partial exposure of the Pt substrate could have led to misleading electrochemical data and therefore had to be avoided.

A recipe for the preparation of Co_3_O_4_(111) on Ir(100) films has been published^32^, but in view of the high OER activity of Ir we decided to avoid this metal as underlayer and developed a new procedure to prepare Co_3_O_4_(111) on Au(111). First, a buffer layer of 2.4 nm of Co was deposited at ~250 K. Following this, 1.6 nm of Co was deposited in 5 × 10^−6^ mbar O_2_ at ~350 K. While maintaining this O_2_ pressure, the sample was heated to 650 K and kept at this temperature while 1.9 nm of Co was deposited. After this, the sample was annealed at 650 K for 10 min. The valve allowing O_2_ into the chamber was closed when the annealing was stopped. The thickness of these films was approximately 10–12 nm.

Co_1+δ_Fe_2−δ_O_4_(111) films were prepared on Pt(111) by following a procedure similar to that used for growing Fe_3_O_4_(111) films on Pt(111)^49^. First, an FeO layer was prepared as detailed above. Following this, Fe and Co were simultaneously deposited at room temperature for ~18 min, followed by annealing in 1 × 10^−5^ mbar O_2_ for at least 30 min at 850 K. This deposition and oxidation cycle was repeated at least three times. Annealing temperatures were limited to 850 K, as for Fe_3_O_4_(111). The stoichiometry of the layers was judged from Fe 2*p* and Co 2*p* XPS spectra. If required, it was adjusted by the addition of iron or cobalt combined with annealing and oxidation. The small differences in the final concentrations of iron and cobalt within these films, denoted Co_1+δ_Fe_2−δ_O_4_(111), did not lead to large differences in the LEED and STM data. These films were 15–20 nm thick. Some STM images and LEED patterns of as-prepared Co_3_O_4_(111), Co_1+δ_Fe_2−δ_O_4_(111), and Fe_3_O_4_(111) are shown in Supplementary Fig. S3.

According to published studies^31,50^ the Fe_3_O_4_(111) surface is terminated with a layer of iron atoms under UHV conditions. In the bulk, these iron ions would be in tetrahedral positions. At the surface, the Co–O coordination in Co_3_O_4_(111) is unsaturated (lower than 4-fold), but the layer is still commonly called ‘tetrahedral’. Based on an IV-LEED study of Co_3_O_4_(111)/Ir(100)^33^, Co_3_O_4_(111) is terminated with a ‘tetrahedral’ Co layer.

### Electrochemical tests

We investigated the electrochemical (EC) performance of the thin films in an electrochemical cell attached to the UHV chamber, allowing transfer in situ without exposure to air. Details of the electrochemistry setup can be found in Supplementary Fig. S12 and discussion. Following electrochemistry, the sample was rinsed using ultrapure water before being reintroduced to the load-lock chamber. The load lock was pumped down to UHV using a turbomolecular pump, before the sample was reintroduced to the UHV analysis chamber for post-electrochemistry analysis.

The following protocol was used for the electrochemical measurements. First, the electrolyte was introduced with the sample at OCV. Then, at OCV + 0.04 V, PEIS was measured, followed by cyclic voltammograms (CVs). The first anodic sweep was from the previous potential, slightly above OCV, to the potential where the current density reached 1 mA/cm^2^. The following CV cycle was between 1 V_RHE_ and the potential corresponding to 1 mA/cm^2^. Then a PEIS spectrum was recorded with the potential at this value. The measured resistance was used for iR correction. Following this, chronoamperometry (CA) data were recorded with the sample at the same potential for approximately 2 h to observe time-dependent reactivity changes. Then, another CV was measured: the first LSV was cathodic, starting from the potential applied during OER down to 1 V_RHE_, followed by an anodic scan to the potential where the current density reached 1 mA/cm^2^. After this, a PEIS spectrum was measured at OER conditions (1 mA/cm^2^). Finally, the seal between the sample and the cell was removed while this potential was still being applied. This was to ensure that the sample did not return to OCV in the electrolyte, as this might induce additional changes at the surface, which might affect the post-reaction surface characterization results. All given potentials and all potential scales are iR-corrected and referenced to the reversible hydrogen electrode (RHE).

In all the electrochemical experiments, we used 0.1 M KOH and calibrated the Ag/AgCl reference electrode against a commercial RHE in the same electrolyte to compensate for any deviations from the nominal pH. For the reference electrode calibration, we measured the open circuit potential between the Ag/AgCl reference electrode and the RHE in the electrolyte after equilibration for at least 15 min. [We note that a potentially wrong calibration of the reference electrode might cause stronger deviations in the current compared to the variations in the OH- concentrations due to errors in the weighting of the chemicals.] We did not specify the pH of the electrolyte in the manuscript to avoid any misleading or wrong statements.

The ohmic resistance of the electrochemical setup was determined from a Nyquist plot obtained via potentiostatic impedance spectroscopy as the high-frequency resistance HFR (HFR = Re(Z) at Im(Z) = 0 Ω).

### Operando Raman measurements

*Operando* Raman measurements were conducted using a Renishaw (InVia Reflex) confocal Raman microscope. The electrochemical measurements were performed in a 0.1 M KOH electrolyte in a home-built spectro-electrochemical cell made of Teflon and controlled by a Biologic SP240 potentiostat. The cell was equipped with a reference electrode (leak-free Ag/AgCl, Alvatek) and a counter electrode (Pt foil). The substrate crystal with the thin film was fixed in place using Kapton tape. The sample was transferred from the UHV chamber to the cell through air. Further details of the *operando* Raman measurements may be found in the SI.

### Supplementary information

Supplementary information

## Data Availability

The source files for all figures can be downloaded from https://isc.archive.fhi.mpg.de/D626. These files contain the relevant experimental data (CV, CA, LEED, XPS, STM and Raman data). Raw data files are available from the corresponding authors upon request.

## References

[1] Cook TR (2010). Solar energy supply and storage for the legacy and nonlegacy worlds. Chem. Rev..

[2] Walter MG (2010). Solar water splitting cells. Chem. Rev..

[3] McCrory CCL (2015). Benchmarking hydrogen evolving reaction and oxygen evolving reaction electrocatalysts for solar water splitting devices. J. Am. Chem. Soc..

[4] O’Sullivan, E. J. M. & Calvo, E. J. in *Comprehensive Chemical Kinetics* **27** (ed Compton, R. G.) Ch. 3, 247–360 (Elsevier, 1988).

[5] Bergmann A (2015). Reversible amorphization and the catalytically active state of crystalline Co3O4 during oxygen evolution. Nat. Commun..

[6] Poulain R, Klein A, Proost J (2018). Electrocatalytic properties of (100)-, (110)-, and (111)-oriented nio thin films toward the oxygen evolution reaction. J. Phys. Chem. C.

[7] Zeng Z (2015). Towards first principles-based prediction of highly accurate electrochemical pourbaix diagrams. J. Phys. Chem. C.

[8] Deng X, Tüysüz H (2014). Cobalt-oxide-based materials as water oxidation catalyst: recent progress and challenges. ACS Catal..

[9] Trasatti S (1980). Electrocatalysis by oxides—attempt at a unifying approach. J. Electroanal. Chem. Interfacial Electrochem..

[10] Corrigan DA (1987). The catalysis of the oxygen evolution reaction by iron impurities in thin film nickel oxide electrodes. J. Electrochem. Soc..

[11] Trotochaud L, Young SL, Ranney JK, Boettcher SW (2014). Nickel–iron oxyhydroxide oxygen-evolution electrocatalysts: the role of intentional and incidental iron incorporation. J. Am. Chem. Soc..

[12] Chanda D, Hnát J, Paidar M, Bouzek K (2014). Evolution of physicochemical and electrocatalytic properties of NiCo_2_O_4_ (AB_2_O_4_) spinel oxide with the effect of Fe substitution at the A site leading to efficient anodic O_2_ evolution in an alkaline environment. Int. J. Hydrog. Energy.

[13] Smith RDL, Prévot MS, Fagan RD, Trudel S, Berlinguette CP (2013). Water oxidation catalysis: electrocatalytic response to metal stoichiometry in amorphous metal oxide films containing iron, cobalt, and nickel. J. Am. Chem. Soc..

[14] Friebel D (2015). Identification of highly active Fe sites in (Ni,Fe)OOH for electrocatalytic water splitting. J. Am. Chem. Soc..

[15] Hunter BM, Winkler JR, Gray HB (2018). Iron is the active site in nickel/iron water oxidation electrocatalysts. Molecules.

[16] Gong L, Chng XYE, Du Y, Xi S, Yeo BS (2018). Enhanced catalysis of the electrochemical oxygen evolution reaction by Iron(III) ions adsorbed on amorphous cobalt oxide. ACS Catal..

[17] Haase FT (2022). Size effects and active state formation of cobalt oxide nanoparticles during the oxygen evolution reaction. Nat. Energy.

[18] Buchner F (2020). Oxygen reduction and evolution on Ni-modified Co_3_O_4_(1 1 1) cathodes for Zn–Air batteries: a combined surface science and electrochemical model study. ChemSusChem.

[19] Faisal F (2018). Atomically defined Co_3_O_4_(111) thin films prepared in ultrahigh vacuum: stability under electrochemical conditions. J. Phys. Chem. C..

[20] Reikowski F (2019). Operando surface X-ray diffraction studies of structurally defined Co_3_O_4_ and CoOOH thin films during oxygen evolution. ACS Catal..

[21] Fester J (2017). Edge reactivity and water-assisted dissociation on cobalt oxide nanoislands. Nat. Commun..

[22] Fester J (2018). The structure of the cobalt oxide/Au catalyst interface in electrochemical water splitting. Angew. Chem. Int. Ed..

[23] Müllner M (2019). Stability and catalytic performance of reconstructed Fe_3_O_4_(001) and Fe_3_O_4_(110) surfaces during oxygen evolution reaction. J. Phys. Chem. C.

[24] Grumelli D (2020). Electrochemical stability of the reconstructed Fe_3_O_4_(001) surface. Angew. Chem. Int. Ed..

[25] Bergmann A (2018). Unified structural motifs of the catalytically active state of Co(oxyhydr)oxides during the electrochemical oxygen evolution reaction. Nat. Catal..

[26] Chung DY (2020). Dynamic stability of active sites in hydr(oxy)oxides for the oxygen evolution reaction. Nat. Energy.

[27] Han S, Liu S, Yin S, Chen L, He Z (2016). Electrodeposited Co-doped Fe_3_O_4_ thin films as efficient catalysts for the oxygen evolution reaction. Electrochim. Acta.

[28] Burke MS, Kast MG, Trotochaud L, Smith AM, Boettcher SW (2015). Cobalt–iron (Oxy)hydroxide oxygen evolution electrocatalysts: the role of structure and composition on activity, stability, and mechanism. J. Am. Chem. Soc..

[29] Horcas I (2007). WSXM: a software for scanning probe microscopy and a tool for nanotechnology. Rev. Sci. Instrum..

[30] McCrory CCL, Jung S, Peters JC, Jaramillo TF (2013). Benchmarking heterogeneous electrocatalysts for the oxygen evolution reaction. J. Am. Chem. Soc..

[31] Ritter M, Weiss W (1999). Fe3O4(111) surface structure determined by LEED crystallography. Surf. Sci..

[32] Heinz K, Hammer L (2013). Epitaxial cobalt oxide films on Ir(100)—the importance of crystallographic analyses. J. Phys. Condens. Matter.

[33] Meyer, W., Biedermann, K., Gubo, M., Hammer, L. & Heinz, K. Surface structure of polar Co_3_O_4_(111) films grown epitaxially on Ir(100)-(1 × 1). *J. Phys.: Condens. Matter* **20**, 265011 (2008). 10.1088/0953-8984/20/26/265011 21694360

[34] Zhao X (2022). Hydrothermal synthesis and formation mechanism of self-assembled strings of CoOOH nanodiscs. Inorg. Chem..

[35] Yang J, Liu H, Martens WN, Frost RL (2010). Synthesis and characterization of cobalt hydroxide, cobalt oxyhydroxide, and cobalt oxide nanodiscs. J. Phys. Chem. C.

[36] Hsu C-S (2017). Valence- and element-dependent water oxidation behaviors: in situ X-ray diffraction, absorption and electrochemical impedance spectroscopies. Phys. Chem. Chem. Phys..

[37] Biesinger MC (2011). Resolving surface chemical states in XPS analysis of first row transition metals, oxides and hydroxides: Cr, Mn, Fe, Co and Ni. Appl. Surf. Sci..

[38] Chen Z (2017). Activity of pure and transition metal-modified CoOOH for the oxygen evolution reaction in an alkaline medium. J. Mater. Chem. A.

[39] Risch M (2015). Water oxidation by amorphous cobalt-based oxides: in situ tracking of redox transitions and mode of catalysis. Energy Environ. Sci..

[40] Song W (2016). Ni- and Mn-promoted mesoporous Co_3_O_4_: a stable bifunctional catalyst with surface-structure-dependent activity for oxygen reduction reaction and oxygen evolution reaction. ACS Appl. Mater. Interfaces.

[41] Nong HN (2020). Key role of chemistry versus bias in electrocatalytic oxygen evolution. Nature.

[42] Faisal F (2018). Electrifying model catalysts for understanding electrocatalytic reactions in liquid electrolytes. Nat. Mater..

[43] Calvillo L (2018). Insights into the durability of Co–Fe spinel oxygen evolution electrocatalysts via operando studies of the catalyst structure. J. Mater. Chem. A.

[44] Liu C, Zou B, Rondinone AJ, Zhang ZJ (2000). Chemical control of superparamagnetic properties of magnesium and cobalt spinel ferrite nanoparticles through atomic level magnetic couplings. J. Am. Chem. Soc..

[45] Zou S (2015). Fe (Oxy)hydroxide oxygen evolution reaction electrocatalysis: intrinsic activity and the roles of electrical conductivity, substrate, and dissolution. Chem. Mater..

[46] Wiegmann T (2022). Operando identification of the reversible skin layer on Co_3_O_4_ as a three-dimensional reaction zone for oxygen evolution. ACS Catal..

[47] Schwarz M (2018). Structure-dependent dissociation of water on cobalt oxide. J. Phys. Chem. Lett..

[48] Sala A (2012). Defects and inhomogeneities in Fe_3_O_4_(111) thin film growth on Pt(111). Phys. Rev. B.

[49] Weiss W, Ranke W (2002). Surface chemistry and catalysis on well-defined epitaxial iron-oxide layers. Prog. Surf. Sci..

[50] Li X (2018). Surface termination of Fe_3_O_4_(111) films studied by CO adsorption revisited. J. Phys. Chem. B.

