# Supplementary material for: Comparative study of Co3O4(111), CoFe2O4(111), and Fe3O4(111) thin film electrocatalysts for the oxygen evolution reaction
Source: Nat Commun. 2023 Aug 8;14:4791. doi: 10.1038/s41467-023-40461-0 (PMC10409724; doi:10.1038/s41467-023-40461-0)
Supplement: Supplementary file 1 — Supplementary information [file 41467_2023_40461_MOESM1_ESM.pdf]

Comparative Study of Co_3O_4(111), $CoFe_2O_4$(111), and Fe_3O_4(111) Thin Film Electrocatalysts for the Oxygen Evolution Reaction

Supplementary information

Earl Matthew Davis[1], Arno Bergmann[1], Chao Zhan[1], Helmut Kuhlenbeck[1,*], Beatriz Roldan Cuenya[1,*]

[1]Department of Interface Science, Fritz-Haber Institute of the Max Planck Society, 14195 Berlin, Germany

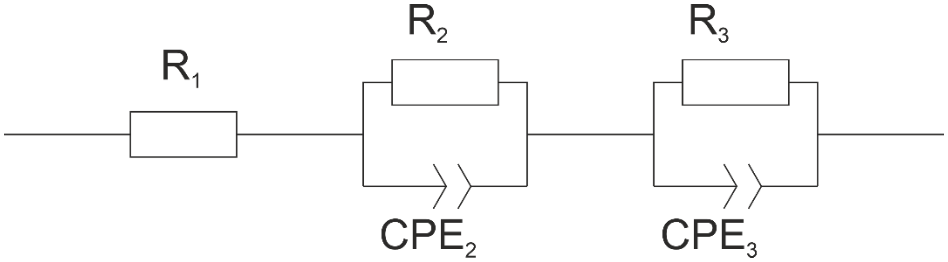

Figure S1 – The double Randles circuit used to model the impedance spectra, incorporating a serial resistor, R_1, and two Randles circuits R_2/CPE_2 and R_3/CPE_3. CPE=constant phase element. R=resistor.

Specific capacitance measurements

Potentiostatic electrochemical impedance spectroscopy (PEIS) was performed on our samples at various stages of the experiment, as outlined in the protocol in the main text. The frequency range was 100 mHz to 500 kHz, with 10 points per decade in logarithmic spacing and an amplitude of 10 mV. The spectra were fitted using a double Randles equivalent circuit with one of the circuits representing the electrolyte-oxide interface and the remaining elements modelling the electrolyte, cabling resistances and electrical resistances within the sample holder. The double-layer capacitance was assumed to be equal to the pseudo-capacitance of the constant phase element of the oxide-electrolyte interface. Values for the various samples are listed in Table S1.

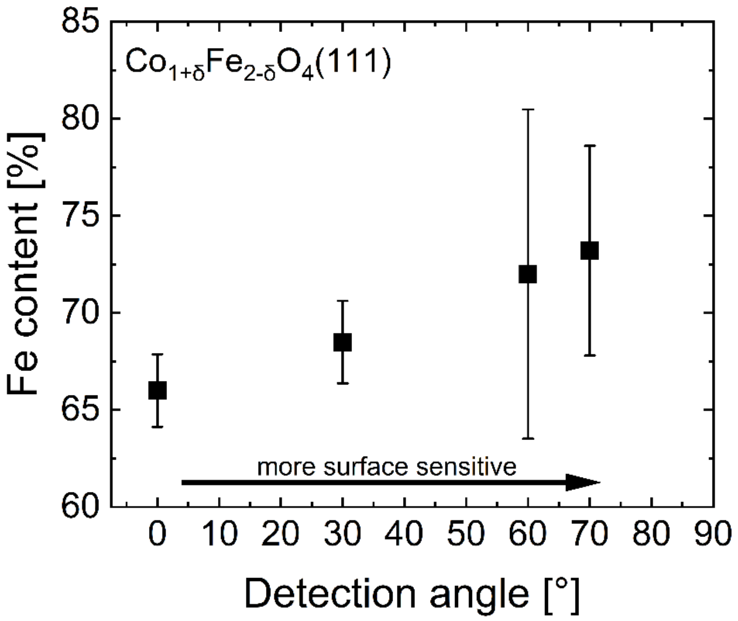

Figure S2 – Fe content relative to the total metal content in a $Co_{1+\delta}Fe_{2-\delta}O_4(111)$, $\delta = 0$, thin film as determined from Fe 2*p* and Co 2*p* peak XPS peak intensities at different photoelectron detection angles relative to the surface normal. These data were acquired on pristine as-prepared films. The error bars are standard deviations computed from several data sets.

Figure S2 shows the change of the Fe 2*p* intensity (relative the sum of the Co 2*p* and Fe 2*p* intensities) as a function of the electron detection angle for the pristine as-prepared $Co_{1+\delta}Fe_{2-\delta}O_4(111)$ samples. The increase of the Fe 2*p* intensity with increasing detection angle indicates that the concentration of Co at the surface is smaller than in the bulk, which is compatible with a mostly iron-terminated surface. In bulk $Co_{1+\delta}Fe_{2-\delta}O_4$, the tetrahedral sites are occupied by Fe^{3+} ions, and XPS does not indicate a change in the Fe oxidation state for the surface layer.

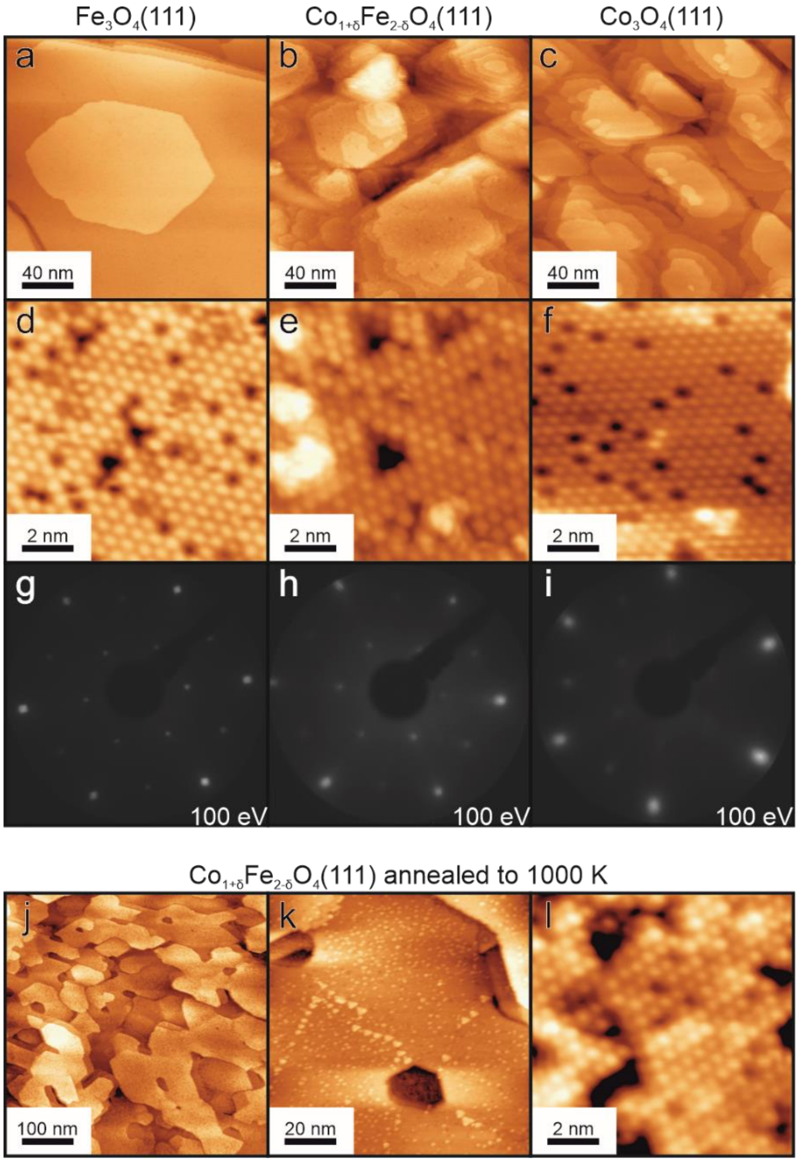

Figure S3: Top panel: structural data for the as-prepared $Fe_3O_4(111)$, $Co_{1+\delta}Fe_{2-\delta}O_4(111)$, and $Co_3O_4(111)$ thin films. (a-c): large scale STM images, (d-f): atomically resolved STM images, (g-i): LEED patterns. STM tunnelling conditions: 2 V, 0.1 nA for (a, c, d, f); -2 V, 0.1 nA for (b, e). The contrast of the LEED images has been modified to increase the brightness of the diffraction features. Bottom panel: STM from a $Co_{1+\delta}Fe_{2-\delta}O_4(111)$ film annealed to 1000 K. Tunnelling conditions: j,l) 2.0 V, 0.020 nA, k) 2.0 V, 0.20 nA.

Structural data of the three oxide films are shown in Figure S3. The large scale STM images (panels a-c) reveal wide terraces (100 nm wide and larger) for $Fe_3O_4(111)$ and somewhat smaller ones for $Co_{1+\delta}Fe_{2-\delta}O_4(111)$ (20-60 nm) and $Co_3O_4(111)$ (10-30 nm).

The atomic scale STM images (Figure S3, d-f) exhibit a certain density of point and extended defects. On $Co_{1+\delta}Fe_{2-\delta}O_4(111)$, triangular pits and islands can be found, with sizes of up to 4 nm across. Paul et al. interpreted similar small triangular islands on $Fe_3O_4(111)$ as adsorbates such as water agglomerates resulting from water in the chamber's residual gas atmosphere[1], which might apply also to the $Co_{1+\delta}Fe_{2-\delta}O_4(111)$ case. The LEED patterns exhibit hexagonal structures as expected for the (111) surfaces of these oxides.

The lower panel of Figure S3 shows STM images from a $Co_{1+\delta}Fe_{2-\delta}O_4(111)$ film that was annealed in 1×10^{-5} mbar O_2 at 1000 K. This produced a highly ordered film with larger terraces more than 100 nm across. Chains of triangular islands are visible across the terraces, which, as mentioned above, may be due to residual water in the UHV chamber.

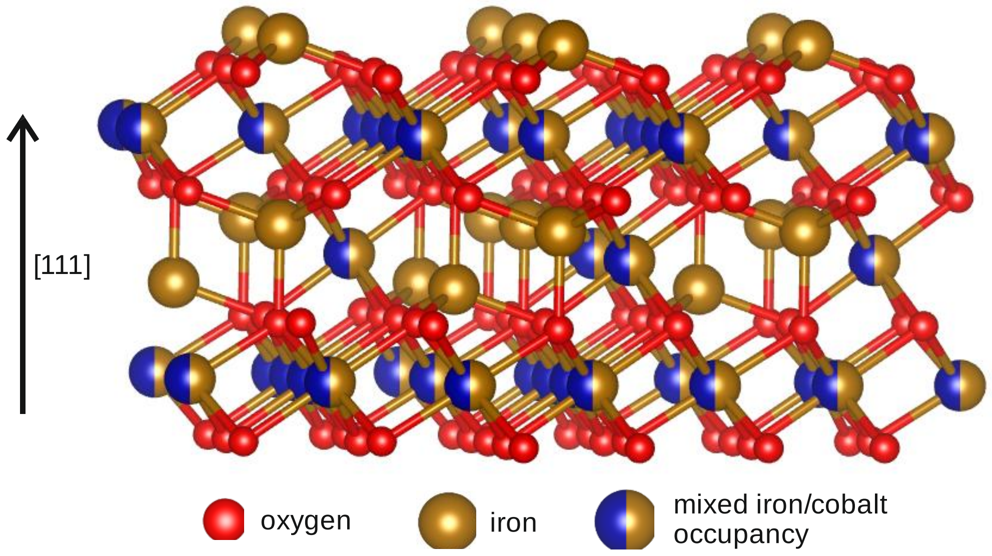

Figure S4: Proposed surface structure of $Co_{1+\delta}Fe_{2-\delta}O_4(111)$ based on STM images and surface-sensitive XPS spectra, assuming an "ideal" bulk-like termination. The surface is terminated with a tetrahedrally-coordinated layer of Fe^{3+} ions (brown). The mixed-colour spheres represent the octahedrally-coordinated cation sites that are equally occupied by Fe^{3+} and Co^{2+} (blue) ions.

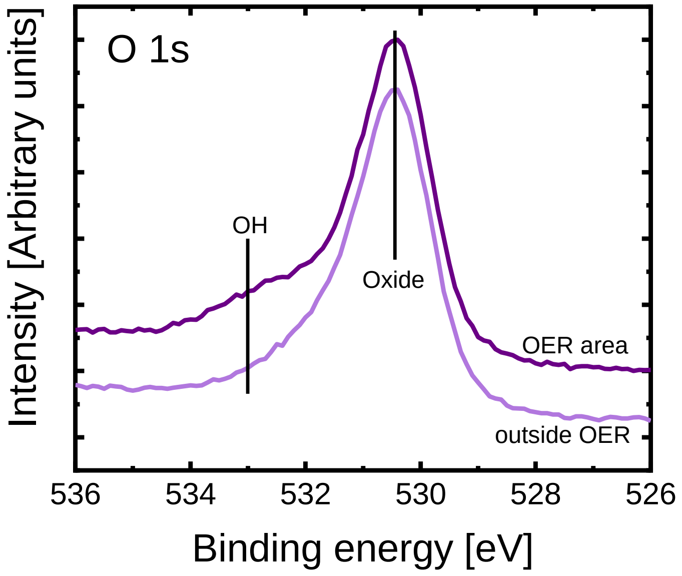

Figure S5 – XPS spectra of the O 1*s* region of the $Fe_3O_4(111)$ film after the OER experiment, comparing the region where OER was performed and an area of the sample that was outside of the area studied electrochemically.

Figure S5 shows that the OH-related O 1*s* shoulder centered around 533 eV is largely due to processes resulting from the exposure of the sample to the electrolyte and the OER.

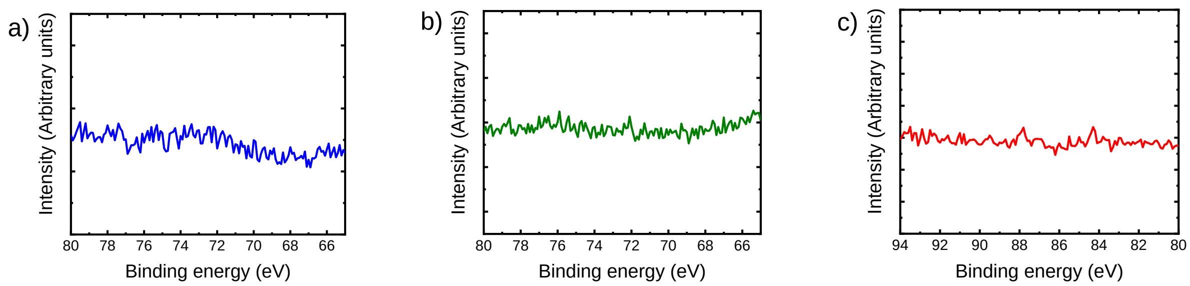

Figure S6 – Post-electrochemistry XPS spectra of the Pt 4f region of the a) $Fe_3O_4(111)$ film, b) $Co_{1+\delta}Fe_{2-\delta}O_4(111)$ film, and c) Au 4f region of the $Co_3O_4(111)$ film.

The data shown in Figure S6 demonstrate that XPS peaks from the substrate material (gold or platinum) are essentially absent after electrochemistry for all three oxide films, so that they do not affect the experimental results.

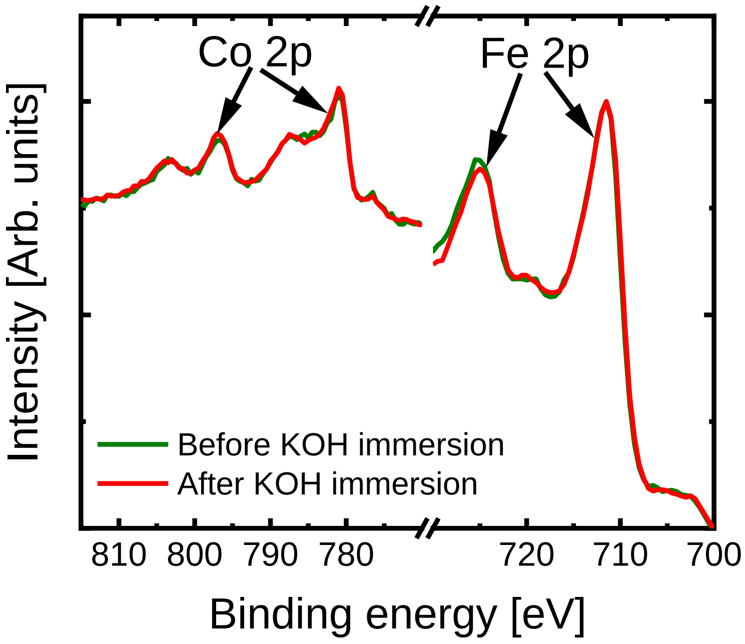

Figure S7 – Co 2*p* and Fe 2*p* XPS spectra for a $Co_{1+\delta}Fe_{2-\delta}O_4$(111) film before and after immersion in a 0.1 M KOH solution for 15 minutes. The spectra show no change in the Fe:Co ratio. The spectra are normalised to equal heights of the Fe 2*p* peaks.

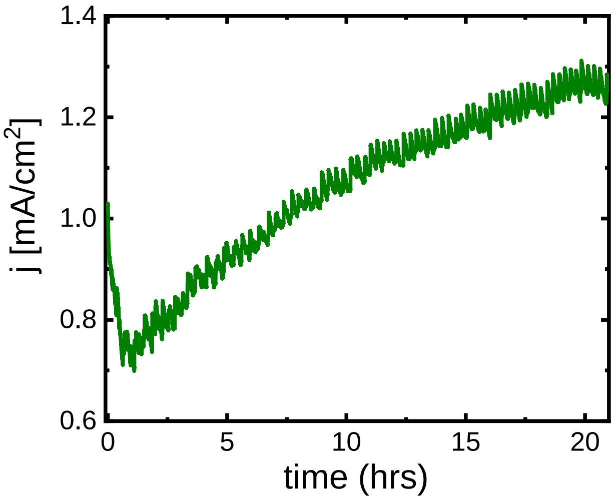

Figure S8 – Chronoamperometry measured on a $Co_{1+\delta}Fe_{2-\delta}O_4(111)$ thin film with a Fe-rich surface layer, measured at 1.755 V_{RHE}.

The initial LSV up to OER conditions has a similar slope and offset as that of the pure $Fe_3O_4(111)$ film. A CA was performed to observe how the current density varied over time, starting at 1 mA/cm^2 (Figure S8). It was found that the film initially behaved similar to the $Fe_3O_4(111)$ film, with an initial increase and then a steady decline in activity to ~0.75 mA/cm^2. However, after approximately 1 hour the current density began again to increase. This gradual increase continued for the following ~18 hours until it reached ~1.25 mA/cm^2, like that of the most active $Co_{1+\delta}Fe_{2-\delta}O_4(111)$ film after 2 hours. Apparently, Co was enriched in the oxyhydroxide layer and the Fe concentration was reduced via Fe dissolution. Surface-sensitive Fe 2*p* and Co 2*p* XPS data (electron detection angle 70°) indicate a decrease of the iron concentration from 78 % to ~50 %.

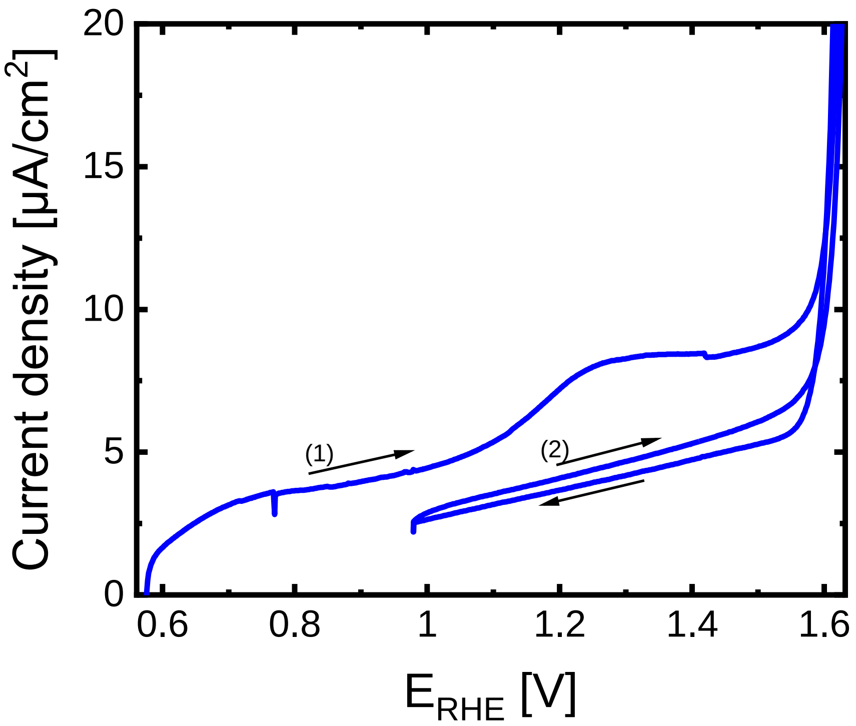

Figure S9 – First CV after film preparation of a $Fe_3O_4(111)$ film. Scan rate 5 mV/s. Electrolyte: 0.1 M KOH. Voltage referred to the reversible hydrogen electrode (RHE).

Figure S9 shows two redox peaks (at ~0.73 and ~1.23 V) in the first anodic sweep for $Fe_3O_4(111)$. The peak at 1.23 V is missing in subsequent sweeps.

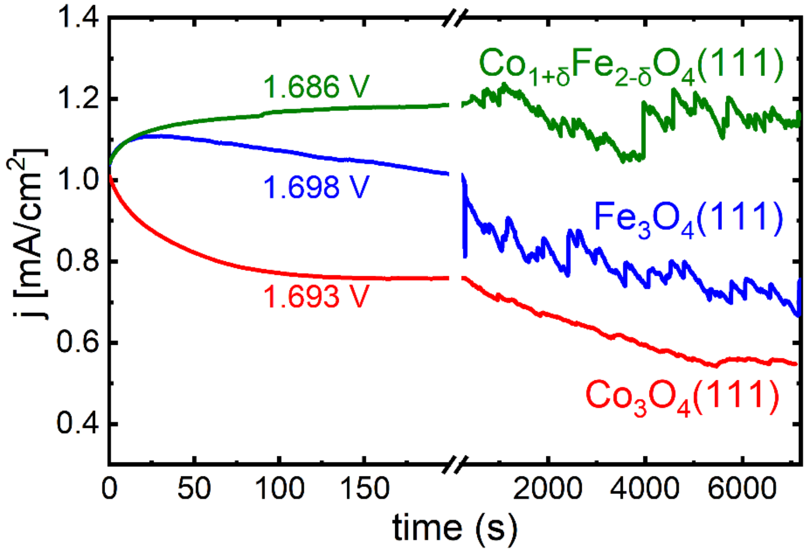

Figure S10 – Time-dependent chronoamperometry measurements of Co_3O_4(111) (red), Fe_3O_4(111) (blue), and $Co_{1+\delta}Fe_{2-\delta}O_4$(111) (green) thin films with the potential set such that the initial current density was 1 mA/cm^2. Potential vs RHE: 1.693 V for Co_3O_4(111), 1.686 V for $Co_{1+\delta}Fe_{2-\delta}O_4$(111), and 1.698 V for Fe_3O_4(111). Data from the most active $Co_{1+\delta}Fe_{2-\delta}O_4$(111) film are shown.

We assign occasional current jumps in the Fe_3O_4(111) and $Co_{1+\delta}Fe_{2-\delta}O_4$(111) CA data in Figure S10 to the formation of bubbles. No such features are observed in the Co_3O_4(111) curve.

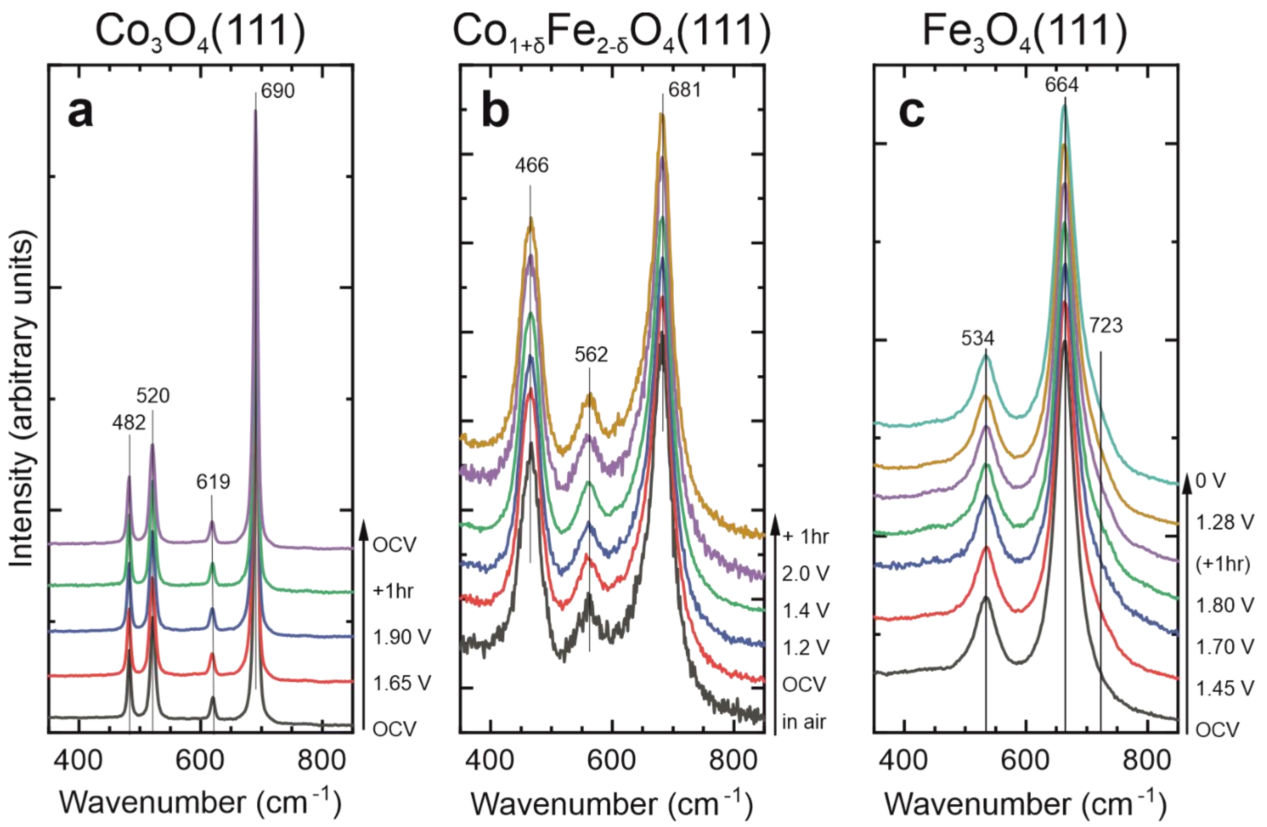

Figure S11 – Operando Raman spectra for a) $Co_3O_4(111)$, b) $Co_{1+\delta}Fe_{2-\delta}O_4(111)$($\delta = 0.04$), and c) $Fe_3O_4(111)$ thin films. A laser wavelength of 532 nm was used for a), c), and 633 nm for b). Potentials are given relative to RHE.

Figure S11 shows *operando* Raman spectra of the 3 oxides for different potentials. The peak positions for the three compositions align well with the reported values.[2] For $Co_3O_4(111)$ and $Co_{1+\delta}Fe_{2-\delta}O_4(111)$ no additional peaks appeared over the course of the measurements. For the $Fe_3O_4(111)$ film, however, a peak appears as a shoulder on the higher-wavenumber side of the main peak at 664 cm^{-1}. There is also a small amount of additional intensity observed at ~350 cm^{-1}. These peaks are known for γ-Fe_2O_3, but not for α-Fe_2O_3 nor for FeOOH, confirming that the $Fe_3O_4(111)$ film is converted to γ-Fe_2O_3 when a positive potential is applied to the sample in the electrolyte. This change begins to occur before the onset of OER, highlighting the instability of $Fe_3O_4(111)$ under these conditions. The decrease of the area of the peak at 534 cm^{-1} (where γ-Fe_2O_3 does not have a strong overlapping peak) was used to calculate the decrease in the signal

from Fe_3O_4 and to estimate how much of the film was converted to γ-Fe_2O_3. After 1 hour at OER conditions, ~25 % of the signal of Fe_3O_4 from the film (initially ~25 nm) had been lost due to conversion to γ-Fe_2O_3.

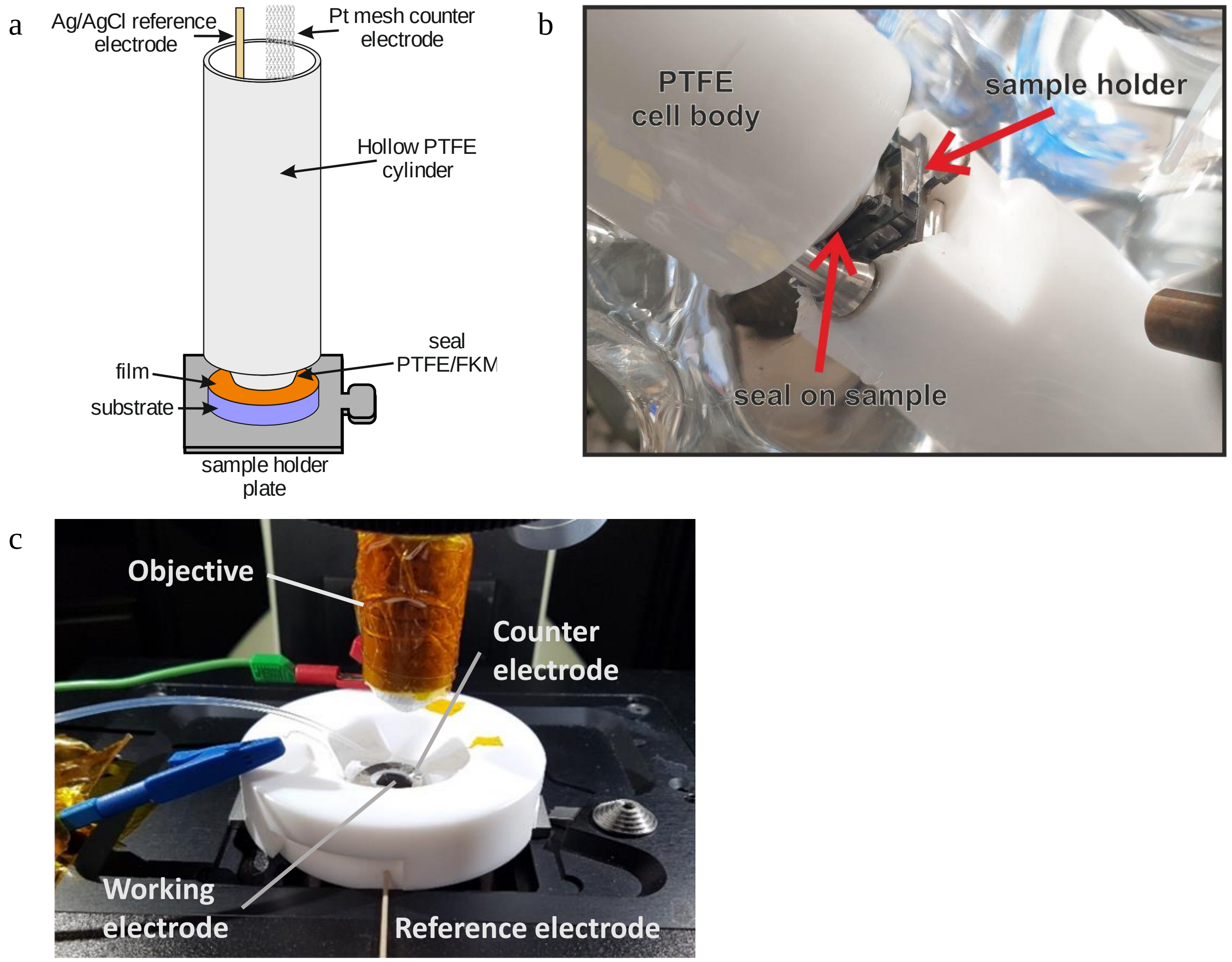

Figure S12 – a) Sketch of the essential parts of the electrochemical (EC) cell at the UHV chamber, b) photo of the cell at the UHV chamber with a sample holder plate and sample, and c) photo of the operando EC sample holder in the Raman system.

Table S1 – Double-layer capacitance of the epitaxial thin film oxides at various stages of the electrochemistry experiments as measured with potentiostatic electrochemical impedance spectroscopy (PEIS).

**Film**	**Double-layer capacitance (mF)**		
	**At OCV+0.04 V**	**Start of OER**	**After 2 hours OER**
$Co_3O_4(111)$	0.022	0.024	0.024
$Co_{1+\delta}Fe_{2-\delta}O_4(111)$	0.023	0.020	0.022
$Fe_3O_4(111)$	0.015	0.025	0.020

Table S2 – Resistances (Ω) for the IR correction of the CVs in Figure 4. The numbers were obtained from PEIS data as explained in the methods section (main text).

**Anode**	**Resistance before OER**	**Resistance after OER**
$Fe_3O_4(111)$	92	88
$Co_3O_4(111)$	84	87
$CoFe_2O_4(111)$	100	108

Table S3 – High-frequency cell resistances (HFR) in Ω for the CV plots in Figure 4. The numbers were obtained from PEIS data as explained in the methods section. We attribute the significant difference between the before- and after-OER values for $Fe_3O_4(111)$ to the oxidation of the iron oxide.

**Anode**	**Resistance before OER**	**Resistance after OER**
$Fe_3O_4(111)$	39	9
$Co_3O_4(111)$	12.6	14
$CoFe_2O_4(111)$	1	4.5

Operando Raman Spectroscopy

Operando Raman measurements were performed using a Renishaw (InVia Reflex) confocal Raman microscope with 532 nm and 633 nm lasers. To perform the operando experiments in the 0.1 M KOH electrolyte, a water immersion objective with a long working distance (Leica microsystems, 63x, numerical aperture of 0.9, working distance of 2.2 mm) was used, prepared as described above. The objective with a long working distance is needed to avoid diffusion hindrance problems during the Raman measurements. The laser power was 9 mW. The acquisition times were between 5 and 50 seconds, and the average of multiple measurements from different areas of the sample was used. The objective was protected from the electrolyte by a Teflon film (DuPont, film thickness of 0.013 mm). A drop of water is used to drive away the air between the film and the objective to match the refractive index, which ensures efficient excitation and collection of the Raman signal. The electrochemical measurements were performed in a home-built spectro-electrochemical cell made of Teflon and controlled by a Biologic SP240 potentiostat. The cell was equipped with a reference electrode (leak-free Ag/AgCl, Alvatek), a counter electrode (Pt foil), and the substrate crystal with the thin film was fixed in place using Kapton tape. The sample was transferred from the UHV chamber to the cell through air.

Electrochemical cell

To ensure that all surfaces in contact with the sample and electrolyte were clean, the electrochemical cell, electrolyte and water bottles, tubing, and metal-free syringes were first immersed in a $KMnO_4$ solution for 24 hours. This ensured that any carbon containing compounds were oxidised. Following this, everything was rinsed with ultrapure water before being immersed in a dilute piranha solution for several minutes. After again rinsing with ultrapure water, the components that can withstand elevated temperatures (i.e., all parts except the FKM seal) were boiled in ultrapure water. Finally, the non-metal parts were placed in a dilute HNO_3 solution overnight to ensure that no metal contamination would be present. A final rinsing in ultrapure water was performed before the cell was inserted into the glass chamber filled with Ar gas where the electrochemical experiments took place.

We investigated the electrochemical (EC) performance of the thin films in an electrochemical cell attached to the UHV chamber, allowing sample transfer *in situ* without exposure to air. The sample was first transferred to a load-lock, which was vented using pure Ar gas, and from there to an attached glass chamber, also filled with Ar gas. Here, the polytetrafluoroethylene (PTFE) electrochemical cell was pressed against the sample, sealing a circular area of the surface with a diameter of 6 mm, see Figure S12. For the Co_3O_4(111) films, the sealing part of the cell was replaced with a fluorocarbon (FKM) piece to avoid damaging the Au(111) substrate. The electrolyte was a 0.1 M KOH solution prepared by dissolving KOH pellets (Sigma Aldrich, 99.99 % purity) in ultrapure water. For the studies on Co_3O_4(111), this was followed by rigorous purification using $Co(OH)_2$ as described by Burke et al.[3] to remove/minimize any Fe contamination. The electrolyte was saturated with Ar, by bubbling the gas through the electrolyte for at least 30 min before the electrolyte was put into the cell. Electrochemical measurements were made using a Biologic SP240 potentiostat, employing a ramp speed of 5 mV/s for the voltammograms. A leakless Ag/AgCl reference electrode was used, and a Pt mesh as counter electrode. The electrolyte was introduced into the cell via a metal-free syringe and sealed tubes to prevent exposure of the electrolyte to air or foreign metals. Following electrochemistry, the sample was rinsed using ultrapure water (which also had Ar bubbled through it), before being reintroduced to the load-lock chamber. The load-lock was pumped down to UHV using a turbomolecular pump, before the sample was reintroduced to the UHV analysis chamber for "quasi *in situ*" post-electrochemistry analysis.

Ultra-high vacuum chamber

An UHV chamber with a base pressure of 4×10^{-11} mbar was used for sample preparation and surface characterization with X-ray photoelectron spectroscopy (XPS), low-energy electron diffraction (LEED), and scanning tunnelling microscopy (STM) at room temperature. The XPS setup comprised an X-ray source with Al and Mg anodes and a hemispherical analyser, all from Omicron GmbH, Germany. Unless stated otherwise, measurements were made at normal emission geometry (0° with respect to the surface normal) using Mg Kα radiation (1253.6 eV). For some measurements the surface sensitivity of XPS was enhanced by measuring at non-normal detection angles. The analyser was operated with a pass energy of 20 eV in constant analyser energy mode.

For binding energy calibration, the Fermi edge and the 4f peaks of a Au(111) crystal were used. A Shirley background was subtracted from the spectra, and peaks were fitted using the pseudo-Voigt functions of the CASA XPS software.[4] An Ar^+ ion gun was employed for sample cleaning. The sample could be heated with the sample set to a positive voltage of up to 1000 V either via thermal radiation or via electron bombardment using a tungsten filament mounted behind the sample. A K-type thermocouple spot-welded to the side of the substrate was employed for sample temperature measurement.

Layer thickness estimation from the damping of LEED spots.

The procedure is based on the damping of the LEED spots by the presence of the layer. For a kinetic energy of 160 eV, the IMFP λ is ~0.6 nm for Co_3O_4. It may be not much different for the hydroxide and the oxyhydroxide. The LEED spot damping may be calculated as $D = e^{-\frac{H}{\lambda}}$ with H being the electron travel distance in the layer. H (and the layer thickness) can be computed from the experimentally observed LEED spot intensity damping. This is rather inaccurate since the arrangement of the oxide surface atoms may be affected by the presence of the layer and the IMFP value does not account for diffuse elastic scattering which will additionally weaken the LEED spots.

Some details of the OER activity calculations

The OER activity numbers are computed for 1 nm^2 electrochemical surface area. We choose to report activity per unit area rather than turnover frequency because, with the transformation of the surface to an oxyhydroxide layer, we cannot be certain of the number of active sites per unit cell. We assume that all of the current is used for O_2 production, i.e., we assume that there is no pseudocapacitive contribution, which might be acceptable in view of the slow voltage ramp speed. The ECSAs were determined from large area (500x500 nm) STM scans as described in the main text.

Computation of sampling depths for XPS

The sampling depth depends on the electron exit angle θ. If the angle is given relative to the surface normal, then the sampling depth D can be computed as $D = \lambda \times \cos(\theta)$, where λ is the inelastic mean free path length of electrons, IMFP. The IMFPs were computed with the IMFP TPP2M program[5] for Co_3O_4. For Fe 2*p*, λ = 12.0 Å and 4.1 Å at electron detection angles of 0° and 70°, respectively. For Co 2*p*, the λ values are 10.9 Å and 3.7 Å. 63% of the XPS intensity stems from a layer with the thickness D, 86% from a 2D thick layer.

Supplementary References

1 Paul, M., Sing, M., Claessen, R., Schrupp, D. & Brabers, V. A. M. Thermodynamic stability and atomic and electronic structure of reduced Fe3O4(111) single-crystal surfaces. *Physical Review B* **76**, 075412 (2007). https://doi.org:10.1103/PhysRevB.76.075412

2 Sanpo, N., Wang, J., Ang, A. S. M. & Berndt, C. C. Influence of the different organic chelating agents on the topography, physical properties and phase of SPPS-deposited spinel ferrite splats. *Applied Surface Science* **284**, 171-178 (2013). https://doi.org:https://doi.org/10.1016/j.apsusc.2013.07.075

3 Burke, M. S., Kast, M. G., Trotochaud, L., Smith, A. M. & Boettcher, S. W. Cobalt–Iron (Oxy)hydroxide Oxygen Evolution Electrocatalysts: The Role of Structure and Composition on Activity, Stability, and Mechanism. *Journal of the American Chemical Society* **137**, 3638-3648 (2015). https://doi.org:10.1021/jacs.5b00281

4 Fairley, N. *et al.* Systematic and collaborative approach to problem solving using X-ray photoelectron spectroscopy. *Applied Surface Science Advances* **5**, 100112 (2021). https://doi.org:https://doi.org/10.1016/j.apsadv.2021.100112

5 Tougaard, S. *QUASES-IMFP-TPP2M Ver.3.0 (downloaded from http://www.quases.com).*
